# Prognostic and immunotherapeutic significance of mannose receptor C type II in 33 cancers: An integrated analysis

**DOI:** 10.3389/fmolb.2022.951636

**Published:** 2022-09-14

**Authors:** Zhixun Zhao, Yanwei Yang, Zheng Liu, Haipeng Chen, Xu Guan, Zheng Jiang, Ming Yang, Hengchang Liu, Tianli Chen, Yibo Gao, Shuangmei Zou, Xishan Wang

**Affiliations:** ^1^ Department of Colorectal Surgery, National Cancer Center/National Clinical Research Center for Cancer/Cancer Hospital, Chinese Academy of Medical Sciences and Peking Union Medical College, Beijing, China; ^2^ Department of Laboratory, National Center for Children’s Health/Beijing Children’s Hospital, Capital Medical University, Beijing, China; ^3^ Department of Thoracic Surgery, National Cancer Center/National Clinical Research Center for Cancer/Cancer Hospital, Chinese Academy of Medical Sciences and Peking Union Medical College, Beijing, China; ^4^ Laboratory of Translational Medicine, National Cancer Center/National Clinical Research Center for Cancer/Cancer Hospital, Chinese Academy of Medical Sciences and Peking Union Medical College, Beijing, China; ^5^ State Key Laboratory of Molecular Oncology, National Cancer Center/National Clinical Research Center for Cancer/Cancer Hospital, Chinese Academy of Medical Sciences and Peking Union Medical College, Beijing, China; ^6^ Central Laboratory, National Cancer Center/National Clinical Research Center for Cancer/Cancer Hospital & Shenzhen Hospital, Chinese Academy of Medical Sciences and Peking Union Medical College, Beijing, China; ^7^ Department of Pathology, National Cancer Center/ National Clinical Research Center for Cancer/ Cancer Hospital, Chinese Academy of Medical Sciences and Peking Union Medical College, Beijing, China

**Keywords:** mannose receptor C type 2 (MRC2), pan-cancer, immunotherapy, immune response, prognosis

## Abstract

**Background:** The type 2 mannose receptor C (MRC2) is involved in tumor biological processes and plays a new role in the remodeling of the extracellular matrix turnover. Previous studies have demonstrated MRC2 expression profiling and prognostic relevance in some tumor types. However, the clinical and immunotherapeutic value of MRC2 in pan-cancers remains controversial. Our study aimed to evaluate MRC2 expression pattern, clinical characteristics and prognostic significance in 33 cancers, explore the relationship between MRC2 and immune-related characteristics, and assess the prediction of MRC2 for the immunotherapeutic response.

**Methods:** Transcriptional and clinical data of 33 cancers were downloaded from The Cancer Genome Atlas database (TCGA) database and two independent immunotherapeutic cohorts were obtained from GSE67501 and the IMvigor210 study. Next, patients stratified by MRC2 expression levels were displayed by Kaplan-Meier plot to compare prognosis-related indexes. Meanwhile, immune infiltrates of different cancers were estimated by tumor immune estimation resources (TIMER) and CIBERSORT. The ESTIMATE algorithm was used to estimate the immune and stromal scores in tumor tissues. MRC2 expression and immunological modulators, including immune inhibitors, immune stimulators, and MHC molecules, were screened through the TISIDB portal. Gene-set enrichment analysis analyses were performed to explore the underlying biological process of MRC2 across different cancers. The immunotherapeutic response prediction was performed in two independent cohorts (GSE78220: metastatic melanoma with pembrolizumab treatment and IMvigor210: advanced urothelial cancer with atezolizumab intervention).

**Results:** MRC2 is expressed differently in many cancers and has been shown to have potential prognostic predicting significance. MRC2 was significantly associated with immune cell infiltration, immune modulators, and immunotherapeutic markers. Notably, the immunotherapeutic response group was associated with lower MRC2 expression in metastatic melanoma and advanced urothelial carcinoma cohort.

**Conclusion:** This study demonstrated that MRC2 could be a prognostic indicator for certain cancer and is critical for tumor immune microenvironments. MRC2 expression level may influence and predict immune checkpoint blockade response as a potential indicator.

## Introduction

More recently, although immune checkpoint blockade therapy is considered a promising strategy for cancers, literature has emerged that less than one-third of the patients who received immunotherapy have significant therapeutic effects (Wang et al., 2019). Except for the antigenicity and mutational burden of cancer, the response to immunotherapy is affected by many factors, such as the composition of the tumor-associated extracellular matrix (ECM) (Madsen and Bugge, 2015). Degradation of the surrounding ECM could promote tumor invasion and destroy the normal tissues. Regarding immunotherapy, ECM could hinder tumor immune infiltration and act as ligands for immune inhibitory receptors (Mariathasan et al., 2018). Consequently, the tumor-associated ECM regulation is expected to provide a novel sight for optimizing the immunotherapeutic strategies and improving the prognosis of cancer (He et al., 2021).

The mannose receptor C type 2 (MRC2), also known as uPARAP/Endo180, plays a pivotal role in the remodeling of the extracellular matrix turnover, such as collagen binding and internalization (Honardoust et al., 2006; Rohani et al., 2014). Meanwhile, MRC2 has an impact on cell migration and invasion involved in tissue repair, cancer progression (Melander et al., 2015; Jurgensen et al., 2020), and more pathological lymphangiogenesis (Engelholm et al., 2001; Durre et al., 2018). It has previously been observed that the expression of MRC2 is aberrantly upregulated in a variety of cancers and associated with poor prognosis, upregulated in including breast cancer, prostate cancer, hepatocellular carcinoma, as well as head and neck cancer (Sulek et al., 2007; Wienke et al., 2007; Kogianni et al., 2009; Palmieri et al., 2013; Gai et al., 2014). However, little systematic research has been focused on the MRC2 expression features and prognosis in pan-cancers. Besides, though extensive research has been carried out on the relationship between immune therapy and ECM, no related study clarified the immune-related characteristics and immunotherapeutic prediction of MRC2 in different cancers.

In our study, we evaluated the MRC2 expression and prognosis-related significance across 33 cancer types based on Cancer Genome Atlas (TCGA) data. Furthermore, the associations between MRC2 and tumor-infiltrating immune cells, immune-related modulators, tumor mutation burden, and microsatellite instability in the tumor microenvironments were analyzed. Additionally, the therapy response with different MRC2 expression levels to immunotherapies for melanoma and urothelial carcinoma was further investigated according to the public immunotherapeutic cohorts.

## Methods and materials

### Data sources

RNA sequencing data and the corresponding clinical information of 33 cancer types were downloaded from TCGA by using the UCSC cancer genome browser (https://tcga. xenahubs.net, accessed April 2020). Totally, 11,007 cases were evaluated in the final analysis and the abbreviations of 33 cancers were summarized in Table 1. Two independent immune therapy cohorts were obtained in this research: The IMvigor210 cohort (advanced urothelial cancer with atezolizumab intervention) was collected from the website based on the Creative Commons 3.0 license (http://research-pub.Gene.com/imvigor210corebiologies) (Mariathasan et al., 2018), and the GSE78220 (metastatic melanoma with pembrolizumab treatment) was downloaded from the Gene Expression Omnibus database (GEO, https://www.ncbi.nlm.nih.gov/geo/).

**TABLE 1:** Abbreviation of 33 human cancers.

Abbreviation	Full name
ACC	Adrenocortical carcinoma
BLCA	Bladder urothelial carcinoma
BRCA	Breast invasive carcinoma
CESC	Cervical squamous cell carcinoma and endocervical adenocarcinoma
CHOL	Cholangiocarcinoma
COAD	Colon adenocarcinoma
DLBC	Lymphoid neoplasm diffuse large B-cell lymphoma
ESCA	Esophageal carcinoma
GBM	Glioblastoma multiforme
HNSC	Head and neck squamous cell carcinoma
KICH	Kidney chromophobe
KIRC	Kidney renal clear cell carcinoma
KIRP	Kidney renal papillary cell carcinoma
LAML	Acute myeloid leukemia
LGG	Brain lower grade glioma
LIHC	Liver hepatocellular carcinoma
LUAD	Lung adenocarcinoma
LUSC	Lung squamous cell carcinoma
MESO	Mesothelioma
OV	Ovarian serous cystadenocarcinoma
PAAD	Pancreatic adenocarcinoma
PCPG	Pheochromocytoma and paraganglioma
PRAD	Prostate adenocarcinoma
READ	Rectum adenocarcinoma
SARC	Sarcoma
SKCM	Skin cutaneous melanoma
STAD	Stomach adenocarcinoma
TGCT	Testicular germ cell tumors
THCA	Thyroid carcinoma
THYM	Thymoma
UCEC	Uterine corpus endometrial carcinoma
UCS	Uterine carcinosarcoma
UVM	Uveal melanoma

### Clinical features and prognosis associated significance of MRC2 in 33 cancers

Gene expression profiles and corresponding clinical information of 33 tumor types was extracted from TCGA. The univariate Cox model was applied to calculate the associations between MRC2 expression levels and patient survival to compare overall survival (OS), disease-free survival (DFS), disease-specific survival (DSS), and progression-free survival (PFS) across the 33 cancer types. Patients stratified by MRC2 expression levels were evaluated by log-rank test and visualized by Kaplan-Meier (KM) curves. MRC2 activity was generated by single-sample gene-set enrichment analysis (ssGSEA), which was utilized to quantify the enrichment scores of immune cells and immune functions for each cancer types. The difference in MRC2 activity between normal and tumor groups was further investigated. To evaluate differences in MRC2 expression at the protein level, IHC images of MRC2 protein expression in normal tissues and tumors tissues, were downloaded from the HPA (http://www.proteinatlas.org/) and analyzed. To evaluate differences in MRC2 expression at the protein level, IHC images of MRC2 protein expression in normal tissues and tumors tissues, were downloaded from the HPA (http://www.proteinatlas.org/) and analyzed. *p* < 0.05 was regarded as a statistical significance.

### MRC2 and immune-associated characteristics in 33 cancers

The tumor immune estimation resources (TIMER, https://cistrome.shinyapps.io/timer/) and CIBERSORT((http://cibersort.stanford.edu/) were carried out to estimate the tumor immune infiltration in different cancers, respectively (Li et al., 2017; Newman et al., 2019). ESTIMATE algorithm was performed to calculate the immune and stromal scores, as well as the correlation with MRC2 expression in tumor tissues. The associations between MRC2 expression and tumor-infiltrating immunocyte related markers were further investigated (Cristescu et al., 2018). The potential relationship between MRC2 expression and immunological modulators, including immune inhibitors, immune stimulators, and MHC molecules, was screened through the TISIDB website (http://cis.hku.hk/TISIDB/index.php). The four most relevant results were then highlighted and presented in plots. The somatic mutation data of all TCGA patients were downloaded (https://tcga.xenahubs.net) and TMB scores and MSI scores were calculated.

### Functional enrichment analysis of MRC2

Subsequently, the expression and activity averages of MRC2 were calculated and ranked for 33 cancers to explore the potential characterization of MRC2 expression and activity. To explore the biological functions of MRC2 in cancers with overall survival prognosis, gene-set enrichment analysis (GSEA) analyzes were performed in BRCA, KIRC, LGG, and UVM, respectively.

### Immunotherapeutic response analysis MRC2

As mentioned above, data obtained from two related independent immunotherapeutic cohorts were analyzed in current study. Patients in complete remission (CR) or partial response (PR) were classified as responders and the remaining cases with stale disease (SD) and progressive disease (PD) were classified as non-response.

### Statistical analysis

In this study, the Wilcox log-rank test was adopted to determine the presence or absence of a markedly increased sum of gene expression z-scores in cancer tissues compared with adjacent normal tissues. Differences in MRC2 expression were also compared in the Kruskal–Wallis test. Survival rates were analyzed using the KM curves, log-rank tests, and Cox proportional hazard regression model models. The Spearman test for correlation analysis. R Language (Version 4.1.1; R Foundation) is available for analysis and the difference of *p* < 0.05 was statistically significant.

## Results

### Clinical profile of MRC2 expression

**FIGURE 1:**
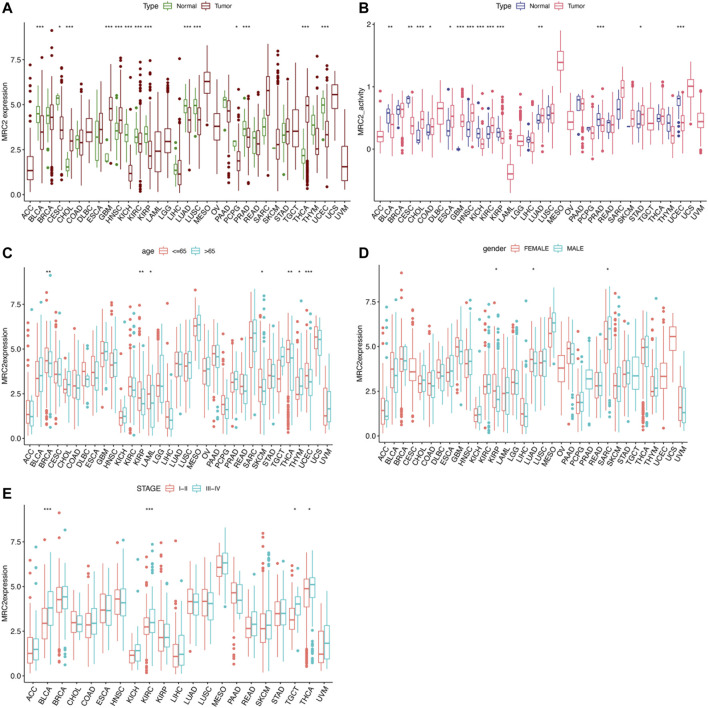
The clinical correlation and activity of MRC2. **(A)** The differential expression analysis between tumor and normal groups of MRC2 in 33 cancers; **(B)** The different activity analysis between tumor and normal groups of MRC2 in 33 cancers; **(C)** The correlation between age and MRC2. **(D)**; The correlation between gender and MRC2; **(E)** The correlation between stage and MRC2. “**” indicates *p* < 0.01 and “***” indicates *p* < 0.001.

As shown in Figure 1A, MRC2 is differentially expressed between tumor and normal tissues in 14 of 33 cancers (Highly expressed in CHOL, GBM, HNSC, and THCA, whereas lowly expressed in BLCA, CESC, KICH, KIRC, KIRP, LUAD, LUSC, PCPG, PRAD, and UCEC). According to the ssGSEA of MRC2 between normal and tumor groups, MRC2 activity was significantly increased in the tumor group of CHOL, ESCA, GBM, HNSC, KIRC, LUAD, and STAD, while decreased in the tumor group of BLCA, CESC, KICH, KIRC, KIRP, PRAD, and UCEC (Figure 1B). Compared to the younger patients (≤65 years old), MRC2 expression decreased in the tumor of elderly patients (>65 years old) in the group of BRCA, KIRP, LAML, SKCM, and UCEC, while the expression pattern was reversed in the THYM group (Figure 1C). With regard to gender, the female group has the higher MRC2 expression in the KRIP and LUAD tumors, while the lower MRC2 level in the SARC tumor (Figure 1D). Besides, MRC2 was positively correlated with the tumor stage of BLCA, KIRC, TCGT, and THCA (Figure 1E). To further explore the differential expression patterns of MRC2 in pan-cancers between tumor and normal tissues, we obtained the related data from the Human Protein Atlas (HPA, https://www.proteinatlas.org). We found that MRC2 was mainly expressed in the tumor stroma, and combined with morphological features, we considered that fibroblasts might be the largest. For tumor cells, we found moderate to strong cytoplasmic positivity was observed in papillary adenocarcinomas of thyroid; a few cases of malignant gliomas, breast, ovarian, endometrial and skin cancers exhibited weak to moderate staining, and remaining malignant cells were mainly negative, which was consistent with our results from pan-cancer analysis (Supplementary Figure S10).

### Correlation of MRC2 expression level and prognosis in 33 cancers

**FIGURE 2:**
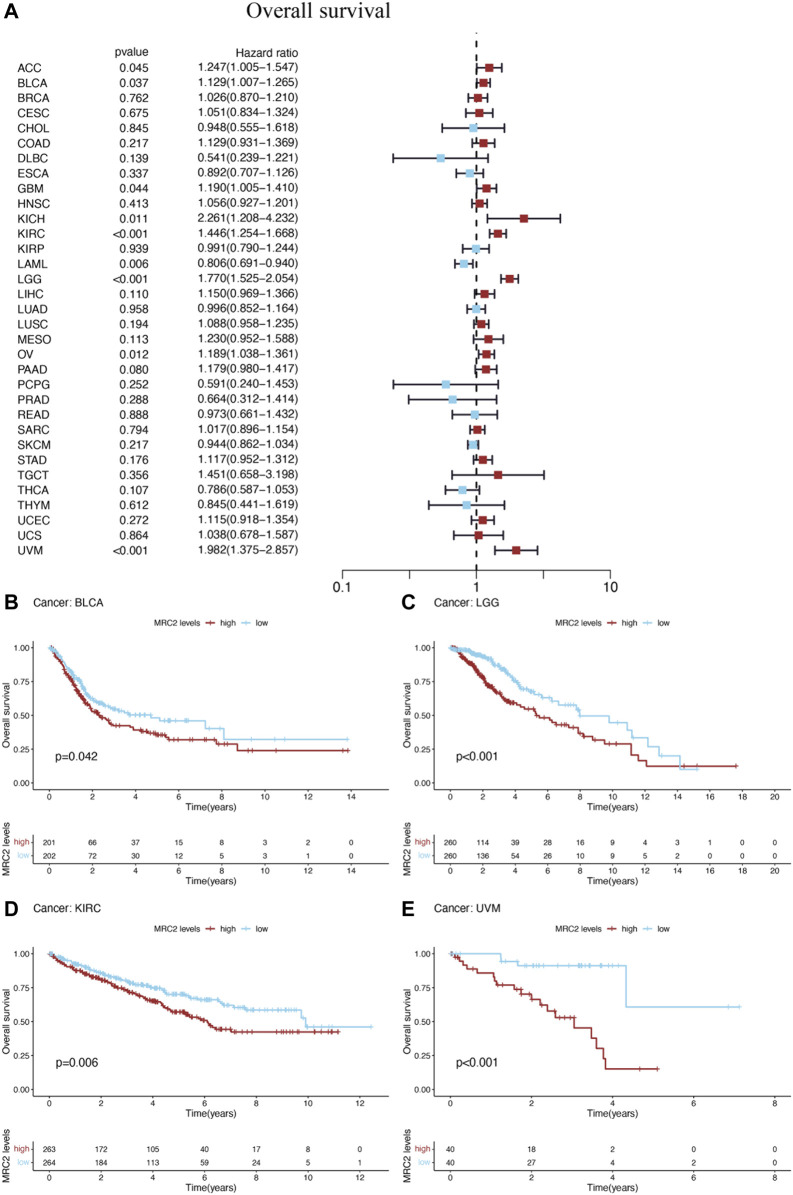
The forest plots of univariate Cox regression analyses for overall survival (OS). **(A)** The highlight items mean that MRC2 expression was significantly correlated with prognosis in these cancer types (*p* < 0.05). Items with hazard ratio greater than 1 indicated that the MRC2 expression was a promoting factor of death. The Kaplan–Meier curves were plotted to visualize the OS of MRC2 expression levels in different cancers **(B–E)**.

Furthermore, high-level MRC2 expression was an unfavorable prognostic indicator for OS in ACC, BLCA, GBM, KICH, KIRC, LAML, LGG, OV, and UVM, as demonstrated in Figure 2A and Supplementary Table S1. In terms of DFS, the higher level of MRC2 was associated with worse outcomes in LGG and PAAD (Supplementary Figure S2A; Supplementary Table S2). Regarding DSS, MRC2 was a risk factor for BLCA, GBM, KICH, KIRC, LGG, OV, PAAD, and UVM (Supplementary Figure S3A; Supplementary Table S3). MRC2 expression was positively correlated with PFS in COAD, KICH, KIRC, PAAD, and UVM and only negatively correlated with DLBC (Supplementary Figure S4A; Supplementary Table S4). Taken all together, MRC2 expression was negatively associated with survival in many tumor types, including ACC, BLCA, GBM, KICH, KIRC, LAML, LGG, OV, and UVM.

The Kaplan–Meier (KM) curves were performed to visualize the prognostic value of MRC2 expression levels in above cancers. High levels of MRC2 expression indicated unfavorable OS in BLCA (*p* = 0.042), LGG (*p* < 0.001), KIRC (*p* = 0.006), and UVM (*p* < 0.001), which was shown in Figures 2B–E. Meanwhile, lower MRC2 was associated with worse DFS in LGG (*p* = 0.013) and PAAD (*p* = 0.036) (Supplementary Figures S2A,C), worse DSS in BLCA (*p* = 0.029), LGG (*p* < 0.001), KIRC (*p* = 0.003), and UVM (*p* < 0.001) (Supplementary Figures S3B–E), worse PFS in COAD (*p* = 0.014), KIRC (*p* < 0.001), LGG (*p* < 0.001), and UVM (*p* < 0.001), and better PFS in only DLBC(*p* = 0.016) (Supplementary Figures S4B–F).

### Correlation between MRC2 expression level and immune-related characteristics

**FIGURE 3:**
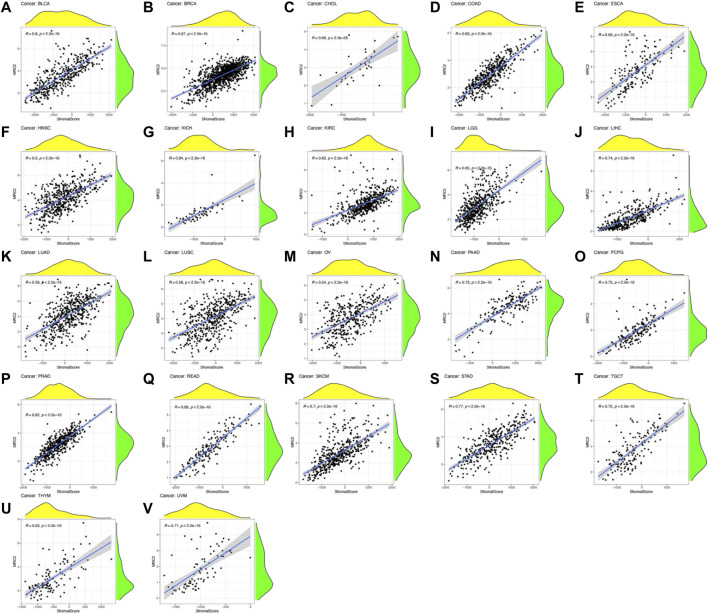
The correlation of MRC2 expression with Stromal Score. The correlation filter was set as *p* < 0.001 and |R| > 0.5 **(A–V)**.

**FIGURE 4:**
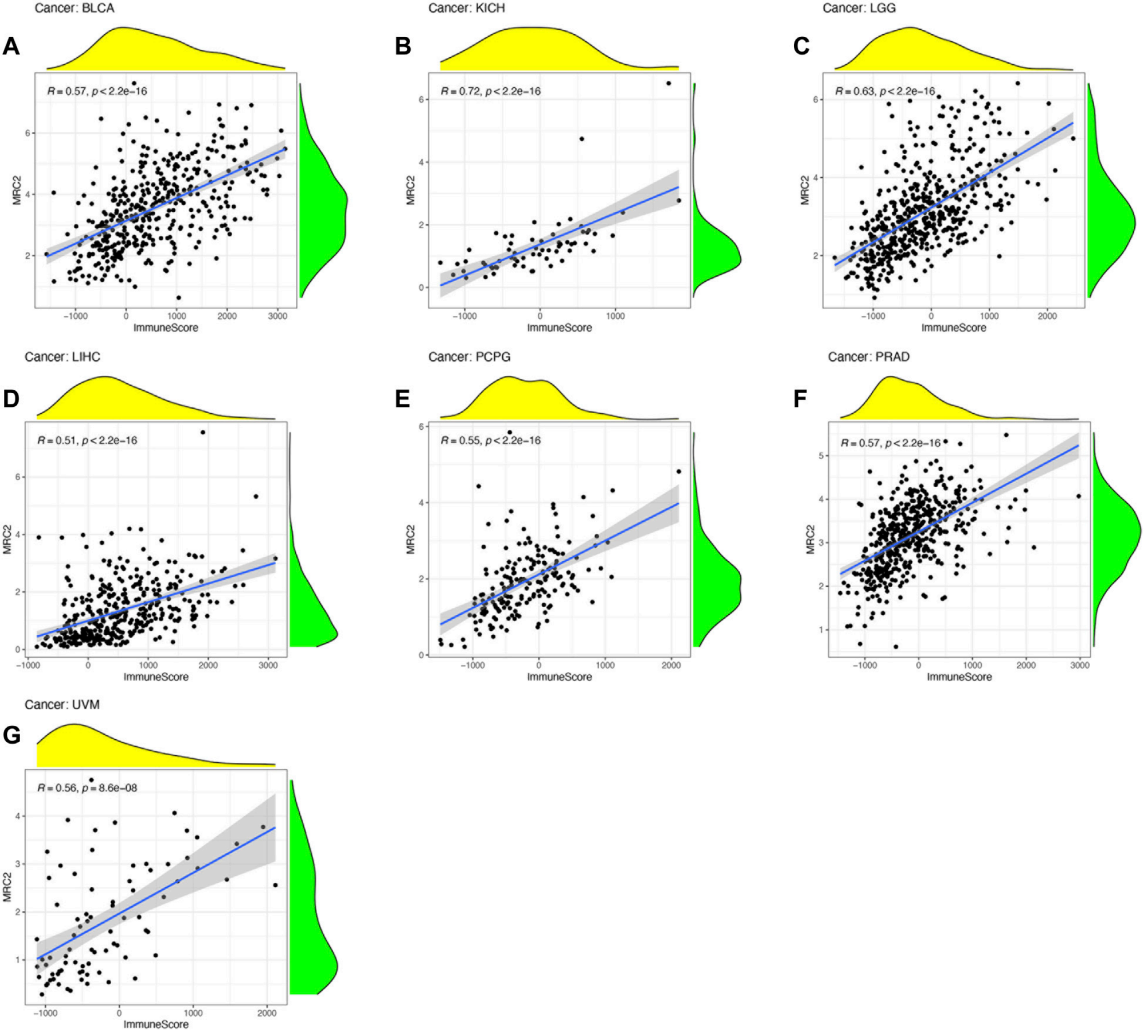
The correlation of MRC2 expression with Immune Score. The correlation filter was set as *p* < 0.001 and |R| > 0.5 **(A–G)**.

ESTIMATE algorithm was used to estimate the stromal score and immune score, with the threshold of *p* < 0.001 and |R| > 0.5. Remarkably, as can be seen from Figure 3, the MRC2 expression was positively correlated with the stromal scores for most the cancer types (BLCA, BRCA, CHOL, COAD, ESCA, HNSC, KICH, KIRC, LGG, LIHC, LUAD, LUSC, OV, PAAD, PCPG, PRAD, READ, SKCM, STAD, TGCT, THYM, and UVM). Relatively, MRC2 is associated with immune scores for BLCA, KICH, LGG, LIHC, PCPG, PRAD, and UVM (Figure 4; Supplementary Table S5). Regarding immune infiltrates (Supplementary Figure S5; Supplementary Table S6), MRC2 expression was positively correlated with the abundance of macrophage M1 and T cells CD8, while negatively with dendritic cells activated in ACC. In TCGT, MRC2 expression was positively correlated with M2 macrophage and negatively associated with B cell naïve and T cells CD4 memory activated. It is also worth mentioning that the MRC2 tended to be correlated to T cells CD8.

**FIGURE 5:**
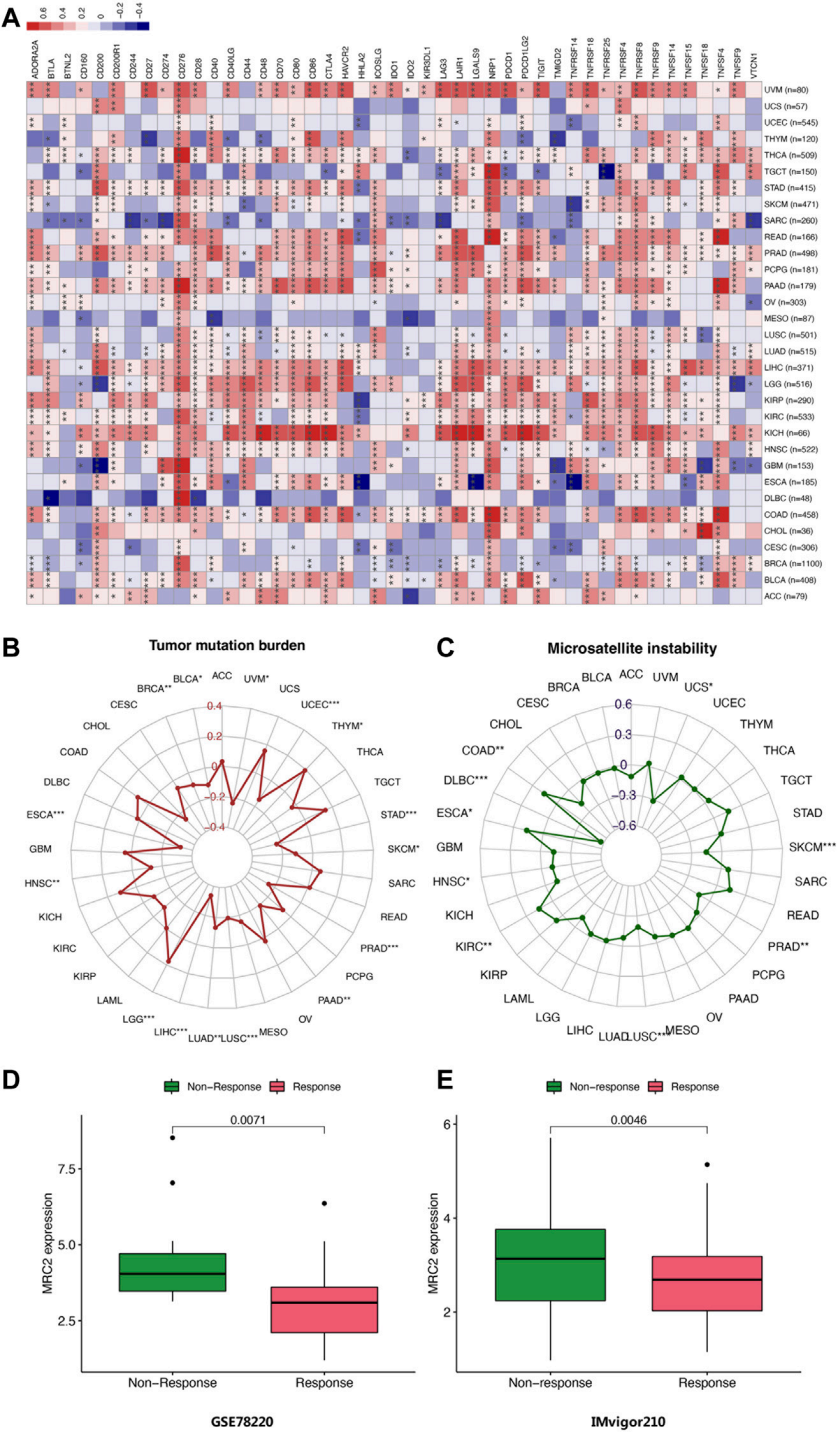
**(A)** Correlation of MRC2 expression with expression of immune checkpoint genes calculated by TIMER. Red indicates positive correlation, whereas blue indicates negative correlation. “*” indicates *p* < 0.05, “**” indicates *p* < 0.01 and “***” indicates *p* < 0.001. **(B)** Correlations between MRC2 expression and TMB. **(B,C)** Correlation between MRC2 and MSI. **(D–E)** Correlations between MRC2 and immunotherapeutic response in immunotherapeutic cohorts.

To further investigate the underlying mechanisms of MRC2 immune inhibition, the TIMER database was taken to compare MRC2 expression with multiple checkpoint markers across different cancer types (Figure 5A). Notably, MRC2 expression in BLCA, COAD, READ, PAAD, and UMV was positively correlated with LAG3, NRP1, CTLA4, PDCD1 (PD-1), CD274 (PD-L1), and PDCD1LG2(PD-L2). To explore the potential of MRC2 to regulate immunomodulators, the relationship between MRC2 and immunomodulators was analyzed by TISIDB. In immune inhibitors, MRC2 was positively associated with PDCD1 in BLCA, CSF1R and PDCD1LG2 in KICH, and TGFBR1 in PRAD (Supplementary Figure S6). In immune stimulators analysis, MRC2 expression was positively correlated with CD86 and TNFSF13B in KICH, TMEM173 in LIHC, and C10orf54 in PRAD (Supplementary Figure S7). Meanwhile, MRC2 expression was positively associated with HLA-DOA, HLA-DPB1, HLA-DQA1 and HLA-DRB1 in KICH (Supplementary Figure S8).

### Analysis of MRC2 immunotherapy response

The correlation between MRC2 expression and TMB as well as MSI was investigated. As demonstrated in Figure 5B, MRC2 has a positive correlation with TMB in LGG and THYM and is negatively correlated with TMB in BLCA, BRCA, ESCA, HNSC, LIHC, LUAD, LUSC, PAAD, PRAD, SKCM, STAD, UCEC, and UVM. In terms of MSI analysis, MRC2 was positively associated with MSI in COAD, ESCA, and KIRC, but negatively correlated with DLBC, HNSC, LUSC, PRAD, SKCM, and USC (Figure 5C). Intriguingly, when analyzing the immunotherapeutic response in a cohort of GSE78220 and IMvigor210, the response group proved the lower MRC2 expression level in metastatic melanoma with pembrolizumab (*p* = 0.071, Figure 5D) and advanced urothelial cancer with atezolizumab (*p* = 0.0046, Figure 5E).

### Functional analysis by GSEA

**FIGURE 6:**
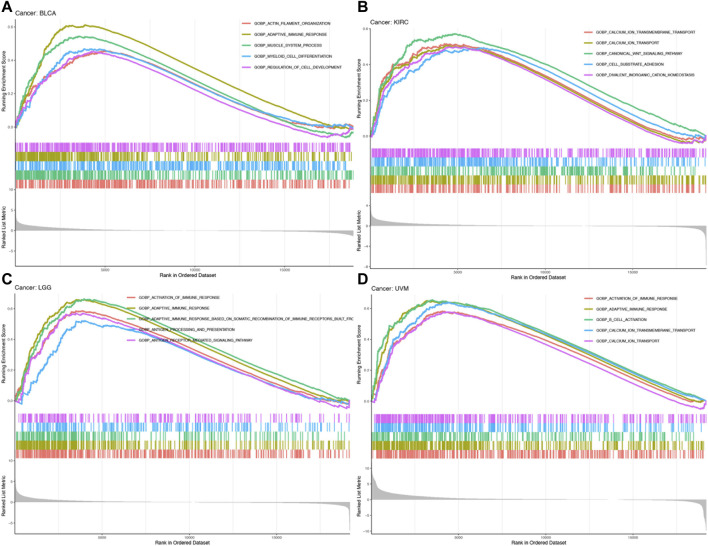
GO enrichment analysis of MRC2 in BLCA **(A)**, KIRC **(B)**, LGG **(C)**, and UVM **(D)**.

To explore the biological functions of MRC2 in cancers with overall survival prognosis, gene-set enrichment analysis (GSEA) analyzes were performed in BRCA, KIRC, LGG, and UVM, respectively. Gene Ontology (GO) analysis indicated that MRC2 was mainly enriched to the activation of the immune response, adaptive immune response, and calcium ion transport (Figure 6; Supplementary Table S9). According to Kyoto Encyclopedia of Genes and Genomes (KEGG) analysis demonstrated in Supplementary Figure S9, MRC2 was enriched in many functions or pathways including chemokine signaling pathway, cytokine receptor interaction, focal adhesion, antigen processing and presentation, and calcium signaling pathway.

## Discussions

There is also increasing research on ECM regulation in cancer immunity, but there is still some confusion. The components of ECM play a critical role in regulating each step of the cancer immunity cycle, which also highlights the potential of targeting tumor-associated ECM to improve cancer immunotherapy. The “hot tumors” characterized by molecular markers of T cell infiltration and immune activation were highly responsive to immunotherapies such as anti-programmed death-ligand 1 (PD-L1)/PD-1 treatment, while “cold tumors” exhibited significant T cell deletion or exclusion (Gajewski, 2015; Zemek et al., 2019). The MRC2, which is involved in homeostatic maintenance and ECM remodeling, plays a role in physiological (embryonic development, wound healing, tissue repair) and pathological conditions (cancer, inflammation) (Lu et al., 2011). Current research has focused on the relationship between MRC2 and tumor immune response and critically analyzed the role of MRC2 in cancer immunity and its potential combination with cancer immunotherapy.

MRC2 expression levels were altered in a variety of cancers. MRC2 expression was highly expressed in the tumor group of CHOL, GBM, HNSC, and THCA, whereas lowly expressed in BLCA, CESC, KICH, KIRC, KIRP, LUAD, LUSC, PCPG, PRAD, and UCEC. There have been accumulating studies reporting that MRC2 expression is increased aberrantly in a variety of cancers. In GBM, MRC2 is upregulated in tumor tissues and mediates tumor cell invasion through collagen-containing stroma (Huijbers et al., 2010; Takahashi et al., 2011). Sulek et al. found that MRC2 expression increased in HNSC tumor compared to adjacent tumors and was positively associated with poor differentiation (Sulek et al., 2007). Previous studies also suggest that in most solid tumors of epithelial origin, expression of MRC2 is reported to be predominantly restricted to cancer-associated fibroblasts (CAFs) with little or no expression by the tumor cells (Sulek et al., 2007; Sulek et al., 2007; Schnack Nielsen et al., 2002; Curino et al., 2005; Koikawa et al., 2018). There is extensive functional evidence implicating CAFs in tumor progression, via their ability to deposit and remodel the extracellular matrix, to secrete pro-tumorigenic factors and by modulating the immune compartment. Besides, there is also evidence that CAFs can play a role in restraining tumor growth, by acting as a desmoplastic barrier to tumor cell invasion and by the recruitment of anti-tumor immune cells. Therefore, MRC2 may play a similar function in tumorigenesis by regulating CAFs.

Previous studies confirmed that downregulation of MRC2 expression reduced the tumor migration and collagen invasion, suggesting active involvement of MRC2 in glioma cell invasion (Huijbers et al., 2010; Takahashi et al., 2011). There is increasing evidence that MRC2 interferes with lymphatic endothelial cells VEGFR-2 and VEGFR-3, which are associated with cancer progression and metastasis to lymph nodes and distant organs (Cady, 2007; Paupert et al., 2011; Durre et al., 2018). Meanwhile, the genetic ablation of MRC2 affects the contractility and viability of cancer associated fibroblasts, limiting tumor growth and metastasis. Based on the above evidence, we suppose that MRC2 expression may contribute to the selection of clinical strategies for certain cancer types. Interestingly, MRC2 suggests poor PFS in multiple tumors, but better PFS in DLBC alone. In most solid tumors of epithelial origin, expression of MRC2 is reported to be predominantly restricted to CAFs with little or no expression by the tumor cells. CAFs play a role in promoting tumorigenesis, metastasis, and drug resistance. Previous studies have confirmed that CAFs generally indicate poor tumor prognosis. However, as a hematological tumor, the roles of MRC2 and CAFs in DLBC may be quite different from those of conventional solid tumors, so there are differences in the predictive prompts.

Next, we investigated the relationship between MRC2 and immune-related characteristics. The MRC2 expression was positively correlated with the stromal scores in 22/33 tumor types, which is consistent with MRC2 as an extracellular matrix remodeling gene. This is possibly because that CAFs play an important role as a component of tumor stroma, and MRC2 expresses predominantly in fibroblasts. In the meantime, MRC2 is correlated to immune scores for BLCA, KICH, LGG, LIHC, PCPG, PRAD, and UVM by ESTIMATE algorithm, which may be due to the fact that CAFs affects the tumor microenvironment in some types tumors. There is also evidence that CAFs can play a role in the recruitment of anti-tumor immune cells (LeBleu and Kalluri, 2018). In addition, the study found that MRC2 expression was correlated with infiltrating levels of macrophage M1 and T cells CD8 in ACC, M2 macrophage, B cell naïve, and T cells CD4 memory activated in TCGT. In addition, the study found that MRC2 expression was correlated with infiltrating levels of macrophage. GSEA also points out that the biological processes of MRC2 in different types of cancer are involved in the activation of immune response and adaptive immune response. Subsequently, using the TIMER and TISIDE databases, we found that MRC2 is associated with important immunomodulatory molecules in multiple tumors. There are a number of known or ongoing immunotherapy-related drug targets, including CD274 (PD1), PDCD1 (PD-L1), PDCD1LG2 (PD-L2), CTLA4, and LAG3. Emerging evidence suggests that components of ECM and its proteolytic remodeling products regulate immune responses and act as immune modulators (Pao et al., 2018). Based on the previous research about the linkage between ECM and the immune microenvironment, the collagens might be the different primary components of ECM between “cold” and “hot” tumors (Pao et al., 2018). Moreover, MRC2 mainly acts on tumor-associated fibroblasts, and affects the characteristics of the tumor microenvironment by regulating extracellular matrix remodeling and secreting cytokines. Previous study confirmed that of epithelial origin, expression of MRC2 is reported to be predominantly restricted to CAFs with little or no expression by the tumor cells in most solid tumors. Likewise, we obtained similar results with the HPA database. However, we found that the expression of MRC2 in certain tumors is related to immune cells, and we consider this to be related to the function of CAFs. In previous studies, CAFs can act on the tumor microenvironment in various ways to produce immune suppression effects, which may include inhibiting the maturation of dendritic cells, abnormal differentiation of T cells, and secreting cytokines to inhibit tumor cell activity. Therefore, the high expression of MRC2 may have an immunosuppressive effect through the function of CAFs, thereby affecting the enrichment of immune cells. All above, targeting MRC2 combined with immune checkpoint blockade therapy may modulate the tumor’s immune status and potentially influence the immunotherapeutic response.

Notably, we investigated the predictive role of MRC2 expression in immunotherapy efficacy in two PD-1 treated immunotherapy cohorts. The results elucidated the potential immunotherapeutic response prediction function of MRC2 in metastatic melanoma and advanced urothelial carcinoma. In previous studies, CAFs can act on the tumor microenvironment in various ways to produce immune suppression effects, which may include inhibiting the maturation of dendritic cells, abnormal differentiation of T cells, and secreting cytokines to inhibit tumor cell activity (LeBleu and Kalluri, 2018). Therefore, the high expression of MRC2 may have an immunosuppressive effect through the function of CAFs, thereby affecting the efficacy of immunotherapy. In follow-up studies, it is necessary to validate the prognostic role of this gene for immune checkpoint therapy in cohorts of other tumor types and larger samples. In follow-up studies, it is necessary to validate the prognostic role of this gene for immune checkpoint therapy in cohorts of other tumor types and larger samples. It is also worth investigating whether inhibition of MRC2 expression can improve the efficacy of immunotherapies. Combination inhibition of MRC2 and immune checkpoints to improve immunotherapeutic efficacy is also a direction for future exploration.

As an article based on public database analysis, this study has certain limitations. The first point is that there is no private data or independent cohort for validation. Second, this study found the associations between MRC2 and CAFs, which may affect the biological functions and characteristics of immunotherapy efficacy. Therefore, it also requires further in-depth study of the function of MRC2 *in vivo* and *in vitro* experiments. Third, all studies in this study were based on bulk sequencing. The research team also consulted the current tumor-related single-cell sequencing database, but it is difficult to meet the evaluation at the pan-cancer level. Therefore, further analysis at the single-cell level in one or several cancer types may be performed in the future, followed by a more in-depth analysis of MRC2.

## Conclusion

In conclusion, we found that MRC2 could be a prognostic indicator for certain cancer and is critical for tumor immune microenvironments. Further exploration of the function of MRC2 might provide influence and predict immune checkpoint blockade response as a potential biomarker.

## Data Availability

The original contributions presented in the study are included in the article/Supplementary Material, further inquiries can be directed to the corresponding authors.

## Supplementary material

Supplementary Figure S1
**(A)** The mean expression of MRC2 in 33 cancers (from high to low); **(B)** The mean activity of MRC2 in 33 cancers (from high to low). “**” indicates p < 0.01 and “***” indicates p < 0.001.

Supplementary Figure S2 The forest plots of univariate Cox regression analyses for disease-free survival (DFS). The highlight items mean that MRC2 expression was significantly correlated with prognosis in these cancer types (*p* < 0.05). Items with hazard ratio greater than 1 indicated that the MRC2 expression was a promoting factor of death. The Kaplan–Meier curves were plotted to visualize the DFS of MRC2 expression levels in different cancers.

Supplementary Figure S3 The forest plots of univariate Cox regression analyses for disease-specific survival (DSS). The highlight items mean that MRC2 expression was significantly correlated with prognosis in these cancer types (*p* < 0.05). Items with hazard ratio greater than 1 indicated that the MRC2 expression was a promoting factor of death. The Kaplan–Meier curves were plotted to visualize the DSS of MRC2 expression levels in different cancers.

Supplementary Figure S4 The forest plots of univariate Cox regression analyses for progression-free survival (PFS). The highlight items mean that MRC2 expression was significantly correlated with prognosis in these cancer types (*p* < 0.05). Items with hazard ratio greater than 1 indicated that the MRC2 expression was a promoting factor of death. The Kaplan–Meier curves were plotted to visualize the PFS of MRC2 expression levels in different cancers.

Supplementary Figure S5 The correlation of MRC2 expression with immune infiltration level in ACC, DLBC, LAML, TGCT, and UVM. The correlation filter was set as *p* < 0.001 and |R| > 0.5.

Supplementary Figure S6 Expression correlation between MRC2 expression and immune inhibitors. The top four immune inhibitors with the correlation coefficient of MRC2 expression level were displayed *via* dot plots.

Supplementary Figure S7 Expression correlation between MRC2 expression and immune stimulators. The top four immune stimulators with the correlation coefficient of MRC2 expression level were displayed *via* dot plots.

Supplementary Figure S8 Expression correlation between MRC2 expression and MHC molecules. The top four MHC molecules with the correlation coefficient of MRC2 expression level were displayed *via* dot plots.

Supplementary Figure S9 KEGG enrichment analysis of MRC2 in BLCA **(A)**, KIRC **(B)**, LGG **(C)**, and UVM **(D)**.

Supplementary Figure S10 Comparison of MRC2 gene expression immunohistochemistry images in normal (left) and tumor (right) between normal and tumor tissues.
