# Supplementary figures and images for: Prognostic and immunotherapeutic significance of mannose receptor C type II in 33 cancers: An integrated analysis
Source: Front Mol Biosci. 2022 Sep 14;9:951636. doi: 10.3389/fmolb.2022.951636 (PMC9519056; doi:10.3389/fmolb.2022.951636)

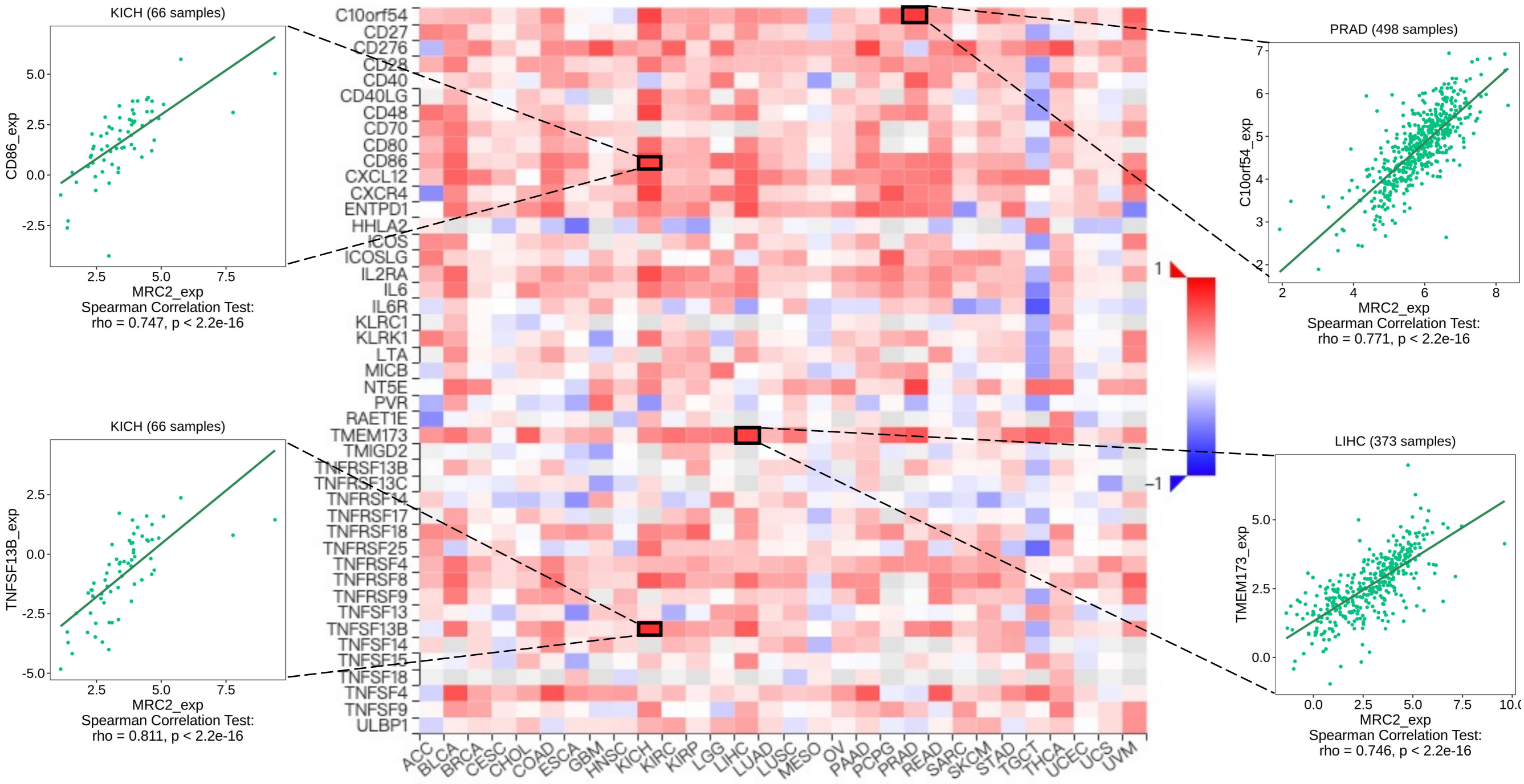

Supplement: Supplementary file 1 [file DataSheet7.PDF]

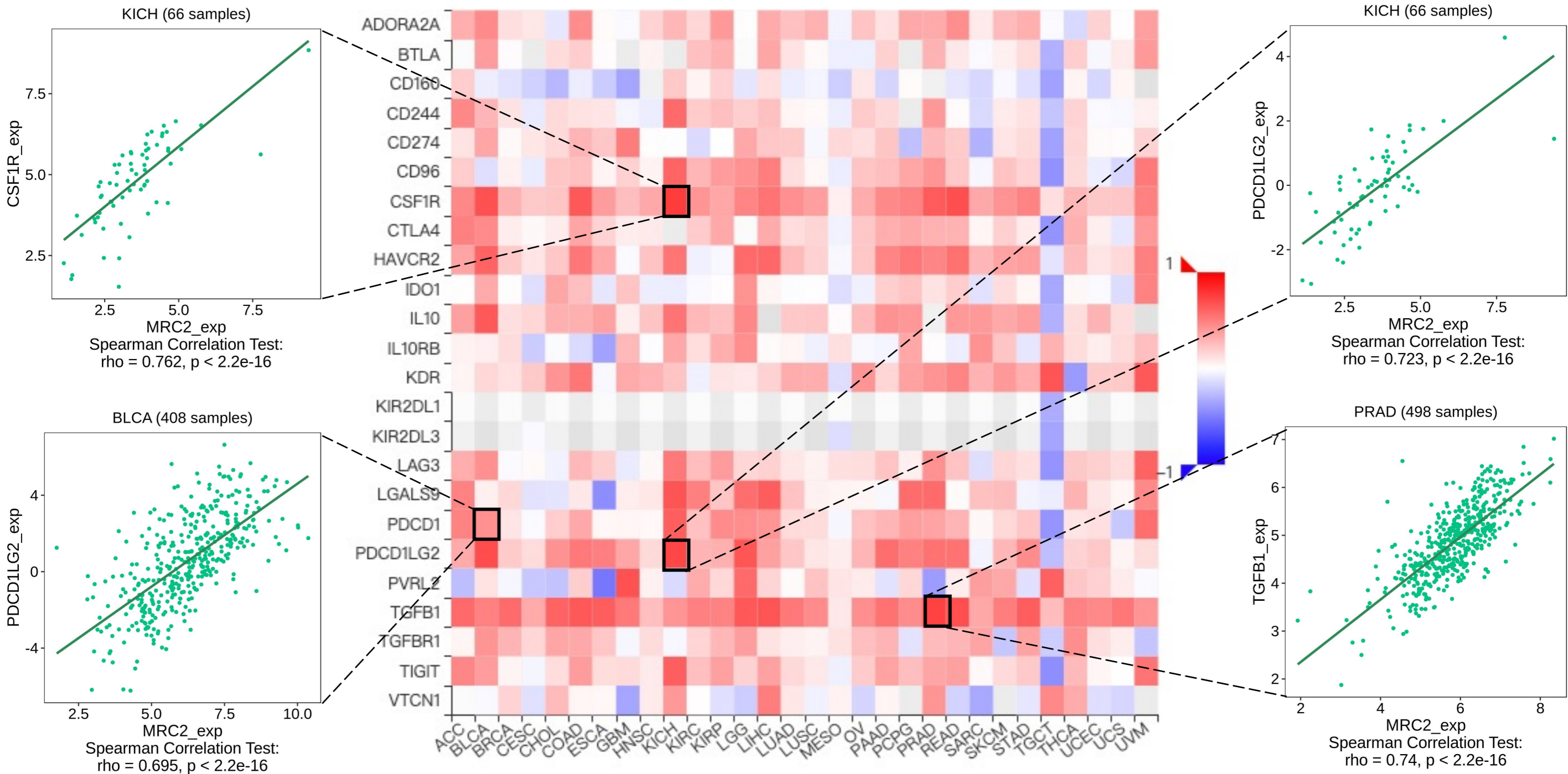

Supplement: Supplementary file 5 [file DataSheet6.PDF]

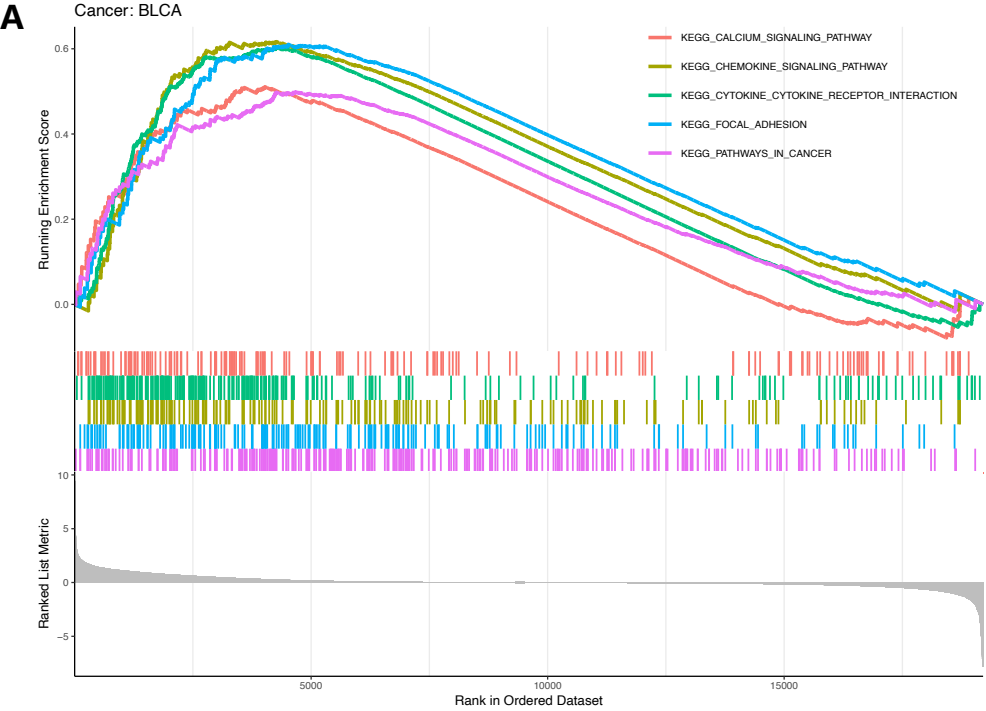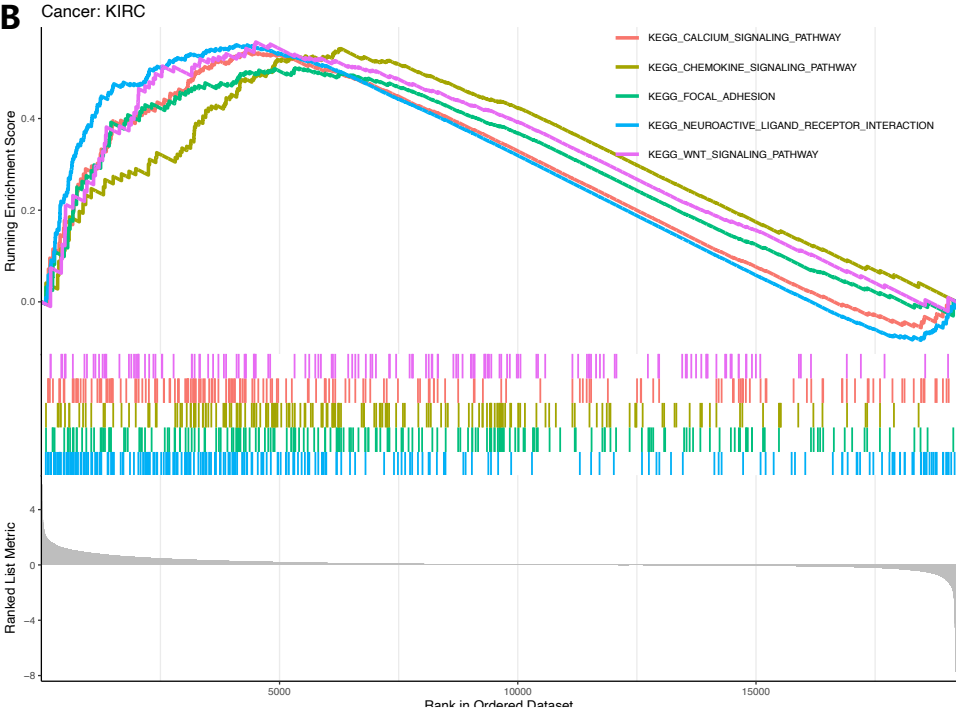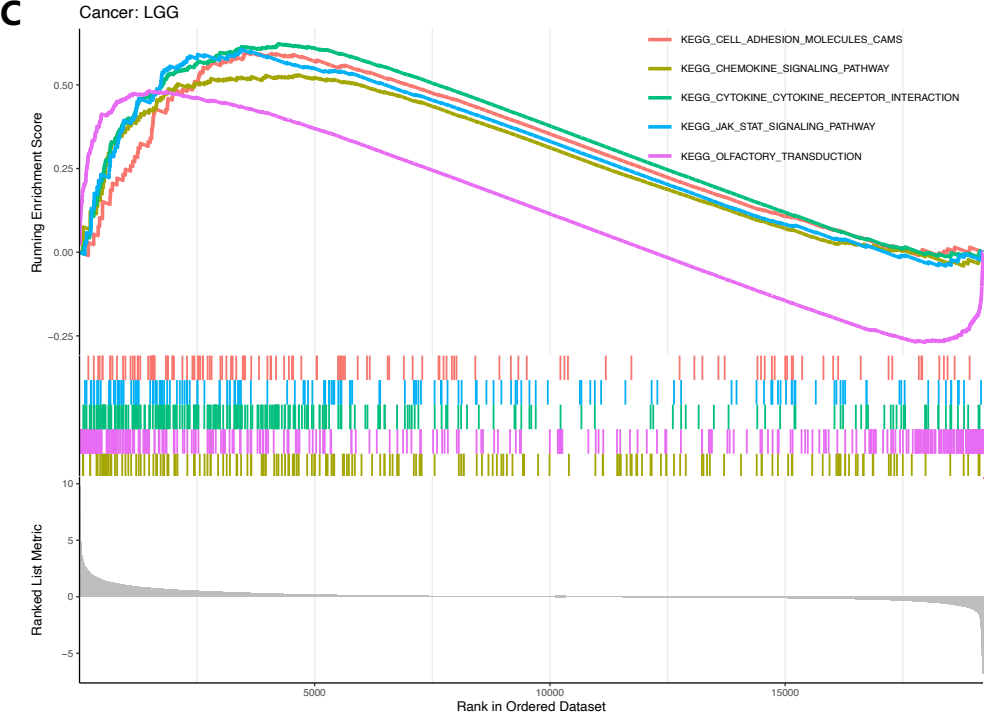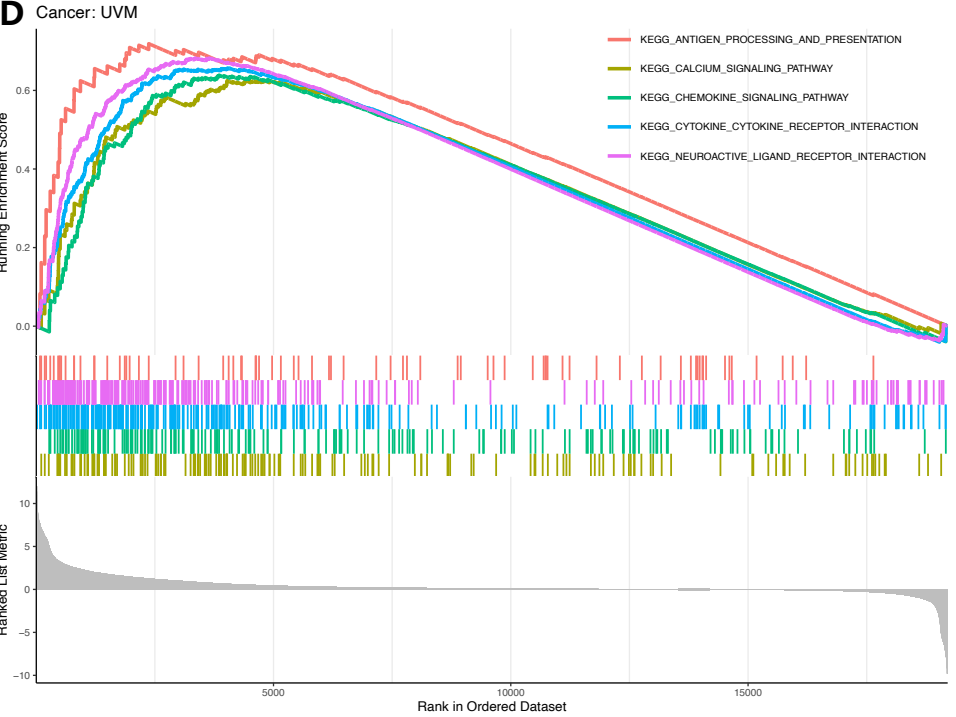

Supplement: Supplementary file 7 [file DataSheet9.PDF]

**A**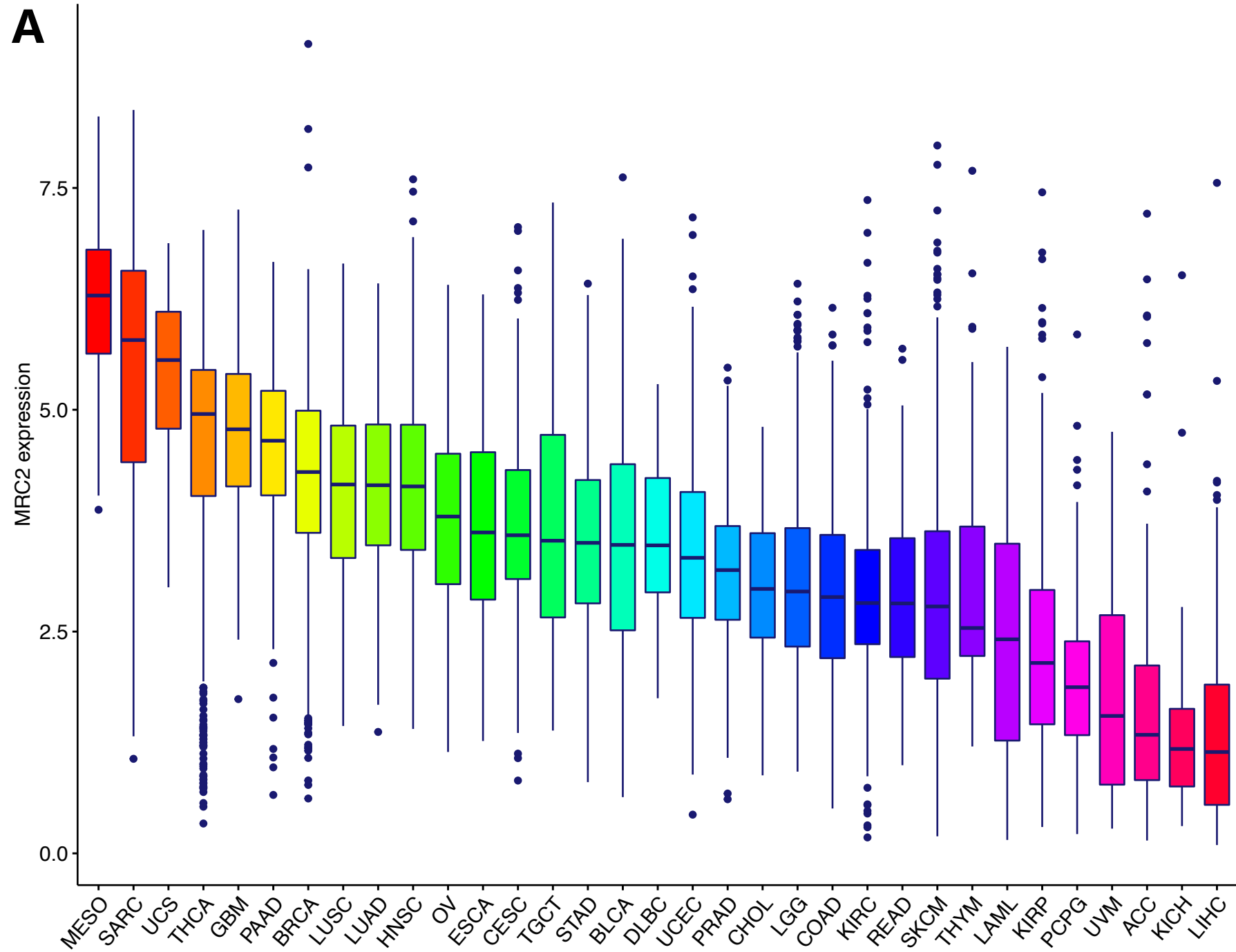**B**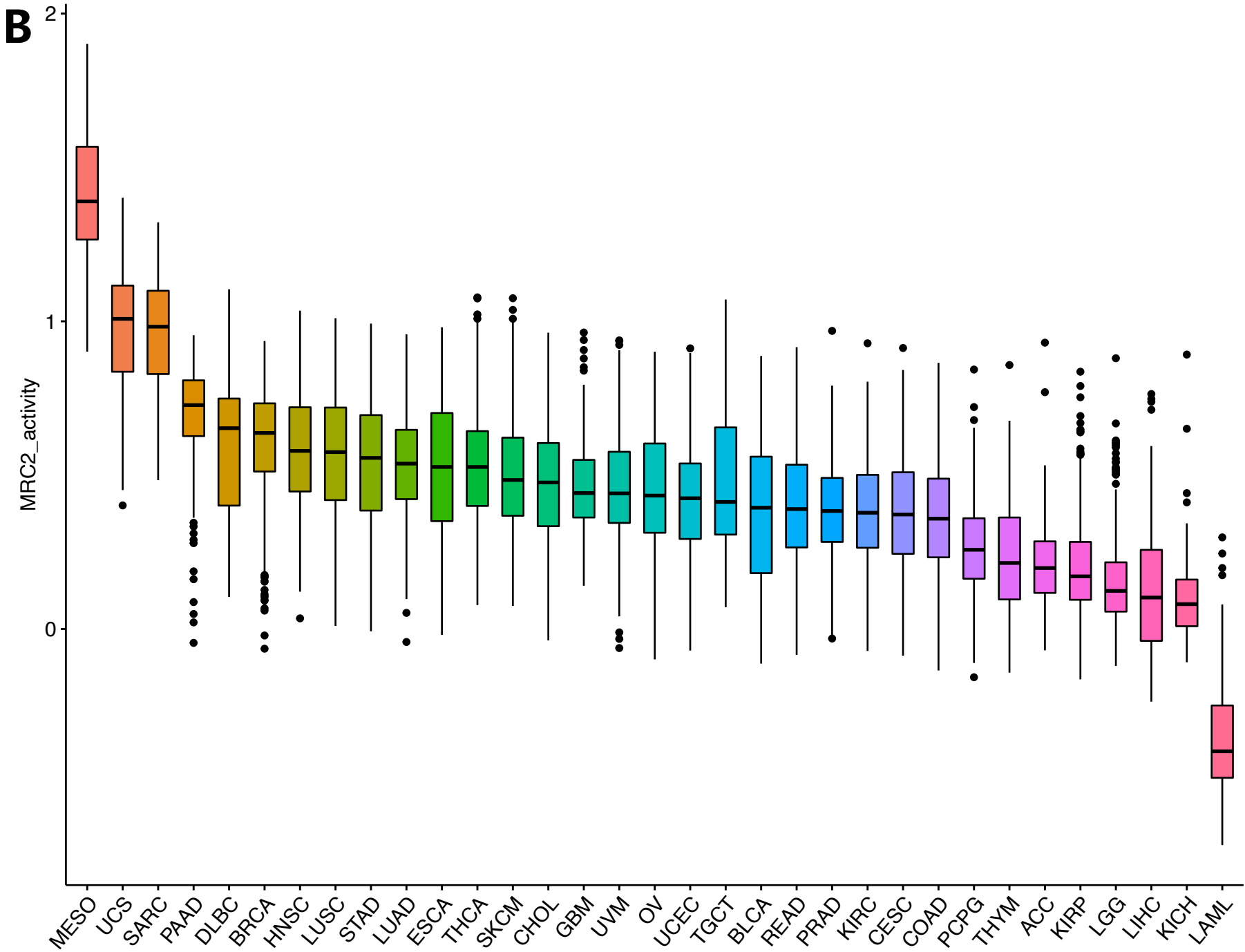

Supplement: Supplementary file 11 [file DataSheet1.PDF]

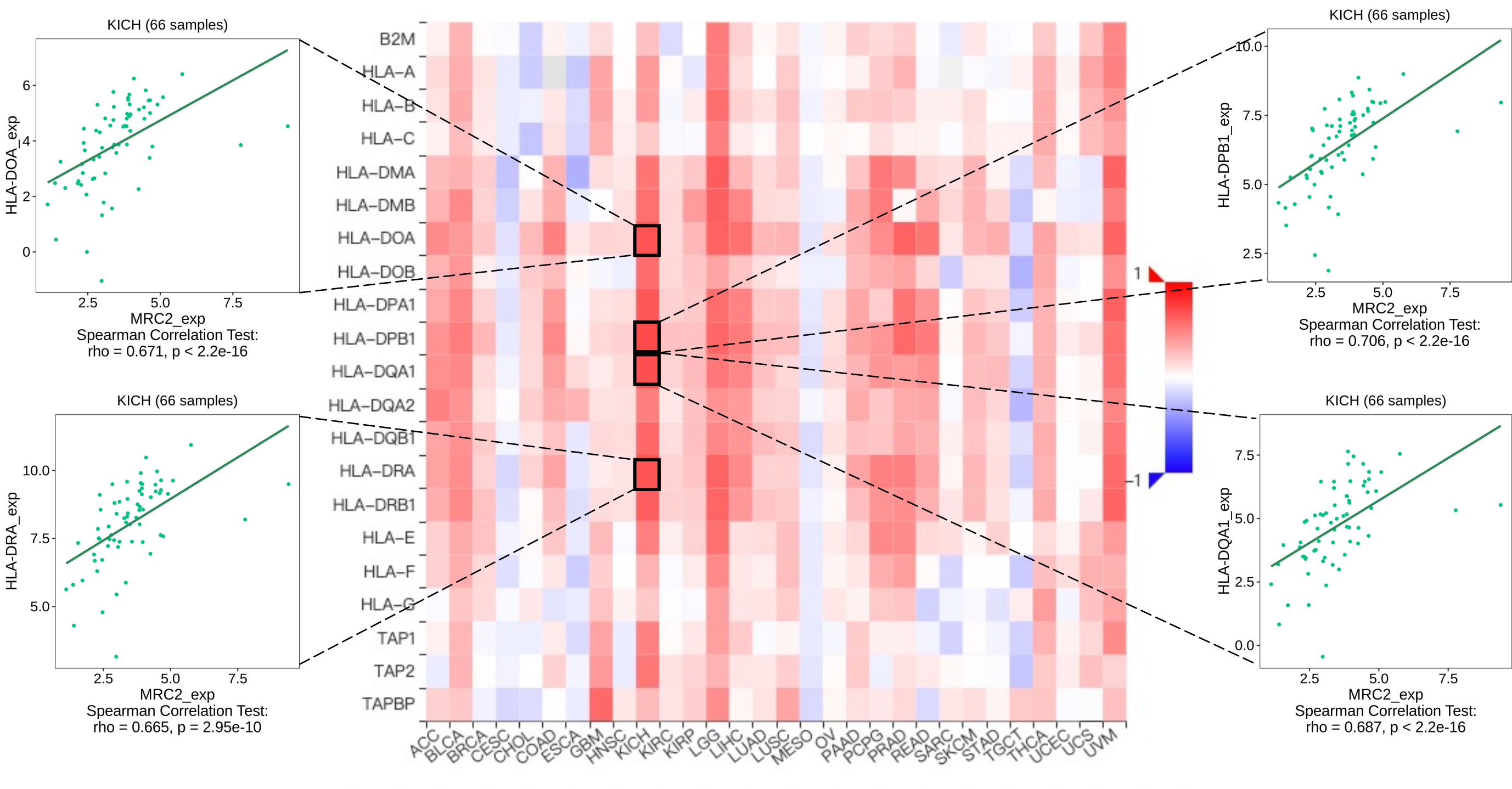

Supplement: Supplementary file 15 [file DataSheet8.PDF]
