# Supplementary material for: Prognostic and immunotherapeutic significance of mannose receptor C type II in 33 cancers: An integrated analysis
Source: Front Mol Biosci. 2022 Sep 14;9:951636. doi: 10.3389/fmolb.2022.951636 (PMC9519056; doi:10.3389/fmolb.2022.951636)
Supplement: Supplementary file 2 [file DataSheet2.PDF]

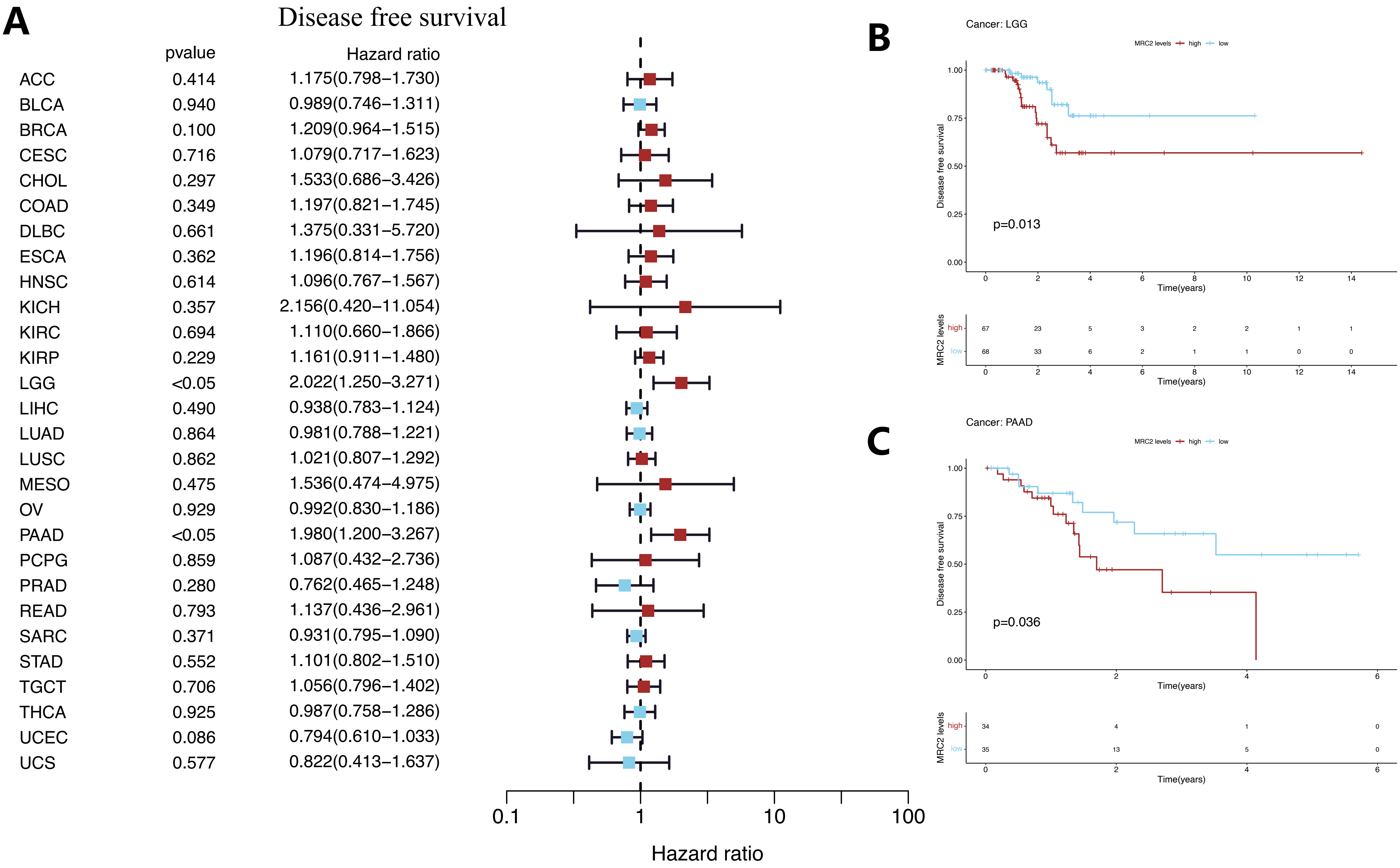
A
Disease free survival
pvalue
Hazard ratio
ACC 0.414 1.175(0.798−1.730)
BLCA 0.940 0.989(0.746−1.311)
BRCA 0.100 1.209(0.964−1.515)
CESC 0.716 1.079(0.717−1.623)
CHOL 0.297 1.533(0.686−3.426)
COAD 0.349 1.197(0.821−1.745)
DLBC 0.661 1.375(0.331−5.720)
ESCA 0.362 1.196(0.814−1.756)
HNSC 0.614 1.096(0.767−1.567)
KICH 0.357 2.156(0.420−11.054)
KIRC 0.694 1.110(0.660−1.866)
KIRP 0.229 1.161(0.911−1.480)
LGG <0.05 2.022(1.250−3.271)
LIHC 0.490 0.938(0.783−1.124)
LUAD 0.864 0.981(0.788−1.221)
LUSC 0.862 1.021(0.807−1.292)
MESO 0.475 1.536(0.474−4.975)
OV 0.929 0.992(0.830−1.186)
PAAD <0.05 1.980(1.200−3.267)
PCPG 0.859 1.087(0.432−2.736)
PRAD 0.280 0.762(0.465−1.248)
READ 0.793 1.137(0.436−2.961)
SARC 0.371 0.931(0.795−1.090)
STAD 0.552 1.101(0.802−1.510)
TGCT 0.706 1.056(0.796−1.402)
THCA 0.925 0.987(0.758−1.286)
UCEC 0.086 0.794(0.610−1.033)
UCS 0.577 0.822(0.413−1.637)
0.1 1 10 100
Hazard ratio
B
Cancer: LGG
MRC2 levels high low
Disease free survival
1.00 0.75 0.50 0.25 0.00
p=0.013
Time(years)
0 2 4 6 8 10 12 14
MRC2 levels
high 67 23 5 3 2 2 1 1
low 68 33 6 2 1 1 0 0
C
Cancer: PAAD
MRC2 levels high low
Disease free survival
1.00 0.75 0.50 0.25 0.00
p=0.036
Time(years)
0 2 4 6
MRC2 levels
high 34 4 1 0
low 35 13 5 0
