# Supplementary material for: Prognostic and immunotherapeutic significance of mannose receptor C type II in 33 cancers: An integrated analysis
Source: Front Mol Biosci. 2022 Sep 14;9:951636. doi: 10.3389/fmolb.2022.951636 (PMC9519056; doi:10.3389/fmolb.2022.951636)
Supplement: Supplementary file 4 [file DataSheet4.PDF]

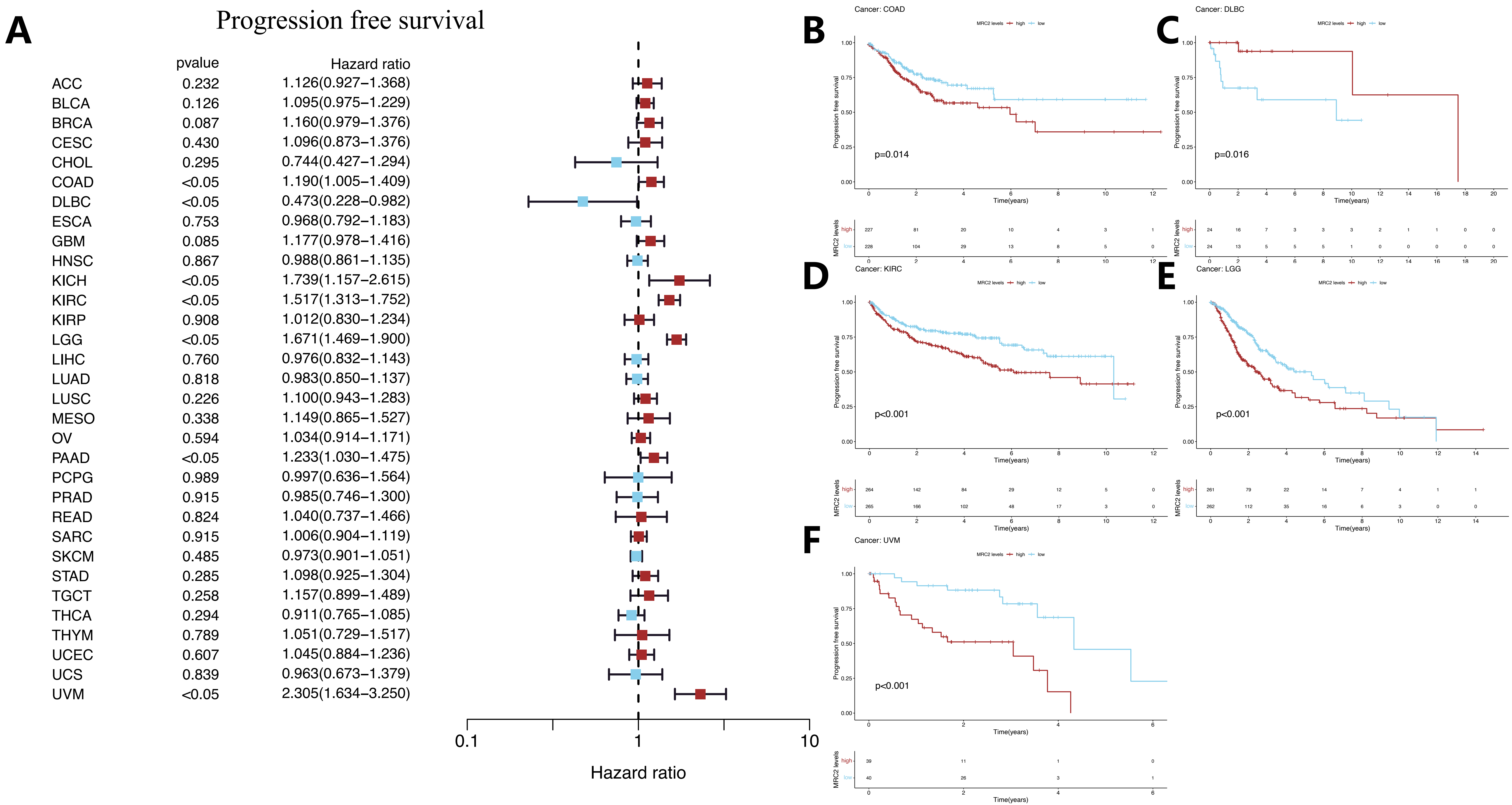
A
Progression free survival
pvalue
Hazard ratio
ACC 0.232 1.126(0.927−1.368)
BLCA 0.126 1.095(0.975−1.229)
BRCA 0.087 1.160(0.979−1.376)
CESC 0.430 1.096(0.873−1.376)
CHOL 0.295 0.744(0.427−1.294)
COAD <0.05 1.190(1.005−1.409)
DLBC <0.05 0.473(0.228−0.982)
ESCA 0.753 0.968(0.792−1.183)
GBM 0.085 1.177(0.978−1.416)
HNSC 0.867 0.988(0.861−1.135)
KICH <0.05 1.739(1.157−2.615)
KIRC <0.05 1.517(1.313−1.752)
KIRP 0.908 1.012(0.830−1.234)
LGG <0.05 1.671(1.469−1.900)
LIHC 0.760 0.976(0.832−1.143)
LUAD 0.818 0.983(0.850−1.137)
LUSC 0.226 1.100(0.943−1.283)
MESO 0.338 1.149(0.865−1.527)
OV 0.594 1.034(0.914−1.171)
PAAD <0.05 1.233(1.030−1.475)
PCPG 0.989 0.997(0.636−1.564)
PRAD 0.915 0.985(0.746−1.300)
READ 0.824 1.040(0.737−1.466)
SARC 0.915 1.006(0.904−1.119)
SKCM 0.485 0.973(0.901−1.051)
STAD 0.285 1.098(0.925−1.304)
TGCT 0.258 1.157(0.899−1.489)
THCA 0.294 0.911(0.765−1.085)
THYM 0.789 1.051(0.729−1.517)
UCEC 0.607 1.045(0.884−1.236)
UCS 0.839 0.963(0.673−1.379)
UVM <0.05 2.305(1.634−3.250)
0.1
1
10
Hazard ratio
B
Cancer: COAD
MRC2 levels high low
Progression free survival
p=0.014
Time(years)
C
Cancer: DLBC
p=0.016
D
Cancer: KIRC
p<0.001
E
Cancer: LGG
p<0.001
F
Cancer: UVM
p<0.001
