# Supplementary material for: Prognostic and immunotherapeutic significance of mannose receptor C type II in 33 cancers: An integrated analysis
Source: Front Mol Biosci. 2022 Sep 14;9:951636. doi: 10.3389/fmolb.2022.951636 (PMC9519056; doi:10.3389/fmolb.2022.951636)
Supplement: Supplementary file 9 [file DataSheet3.PDF]

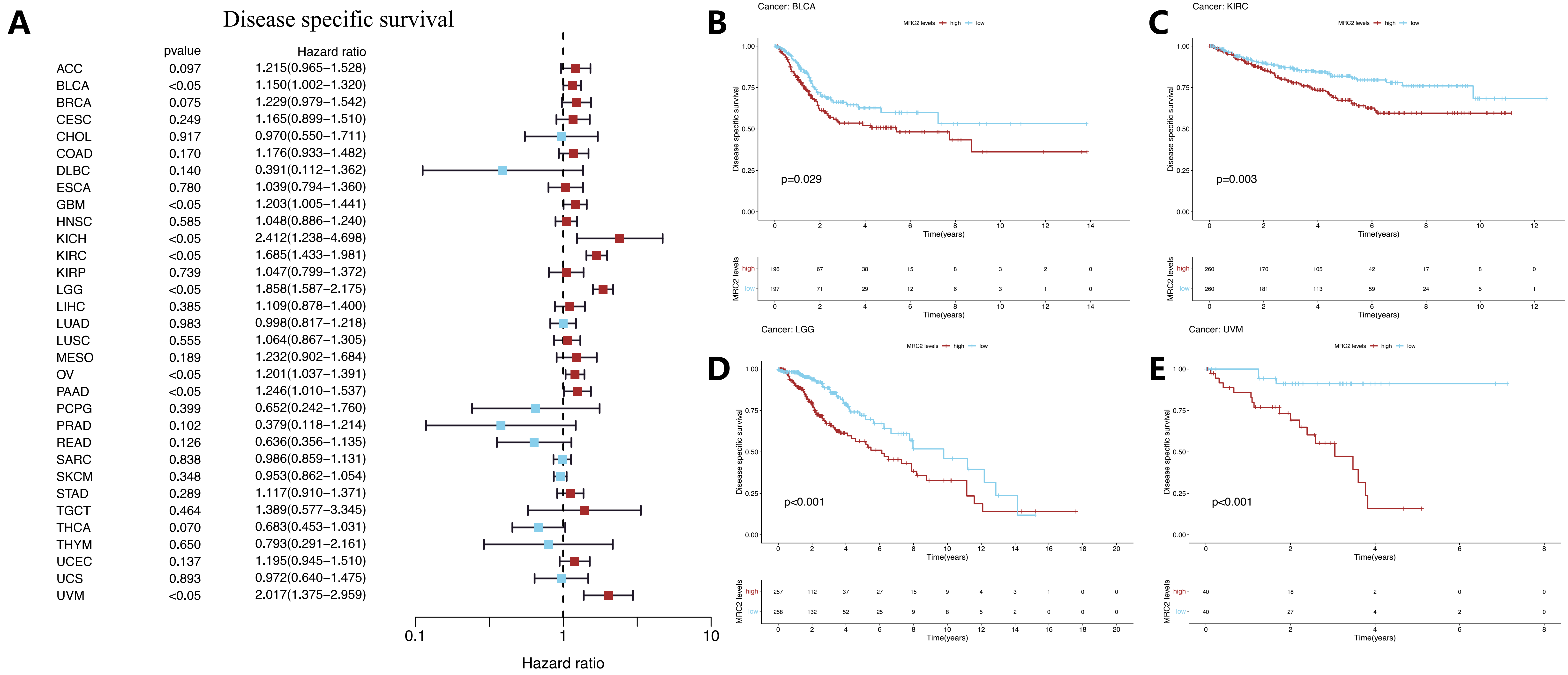
A
Disease specific survival
pvalue
Hazard ratio
ACC 0.097 1.215(0.965–1.528)
BLCA <0.05 1.150(1.002–1.320)
BRCA 0.075 1.229(0.979–1.542)
CESC 0.249 1.165(0.899–1.510)
CHOL 0.917 0.970(0.550–1.711)
COAD 0.170 1.176(0.933–1.482)
DLBC 0.140 0.391(0.112–1.362)
ESCA 0.780 1.039(0.794–1.360)
GBM <0.05 1.203(1.005–1.441)
HNSC 0.585 1.048(0.886–1.240)
KICH <0.05 2.412(1.238–4.698)
KIRC <0.05 1.685(1.433–1.981)
KIRP 0.739 1.047(0.799–1.372)
LGG <0.05 1.858(1.587–2.175)
LIHC 0.385 1.109(0.878–1.400)
LUAD 0.983 0.998(0.817–1.218)
LUSC 0.555 1.064(0.867–1.305)
MESO 0.189 1.232(0.902–1.684)
OV <0.05 1.201(1.037–1.391)
PAAD <0.05 1.246(1.010–1.537)
PCPG 0.399 0.652(0.242–1.760)
PRAD 0.102 0.379(0.118–1.214)
READ 0.126 0.636(0.356–1.135)
SARC 0.838 0.986(0.859–1.131)
SKCM 0.348 0.953(0.862–1.054)
STAD 0.289 1.117(0.910–1.371)
TGCT 0.464 1.389(0.577–3.345)
THCA 0.070 0.683(0.453–1.031)
THYM 0.650 0.793(0.291–2.161)
UCEC 0.137 1.195(0.945–1.510)
UCS 0.893 0.972(0.640–1.475)
UVM <0.05 2.017(1.375–2.959)
0.1
1
10
Hazard ratio
B
Cancer: BLCA
MRC2 levels high low
Disease specific survival
p=0.029
Time(years)
MRC2 levels
high 196 67 38 15 8 3 2 0
low 197 71 29 12 6 3 1 0
C
Cancer: KIRC
MRC2 levels high low
Disease specific survival
p=0.003
Time(years)
MRC2 levels
high 260 170 105 42 17 8 0
low 260 181 113 59 24 5 1
D
Cancer: LGG
MRC2 levels high low
Disease specific survival
p<0.001
Time(years)
MRC2 levels
high 257 112 37 27 15 9 4 3 1 0 0
low 258 132 52 25 9 8 5 2 0 0 0
E
Cancer: UVM
MRC2 levels high low
Disease specific survival
p<0.001
Time(years)
MRC2 levels
high 40 18 2 0 0
low 40 27 4 2 0
