# Supplementary material for: Prognostic and immunotherapeutic significance of mannose receptor C type II in 33 cancers: An integrated analysis
Source: Front Mol Biosci. 2022 Sep 14;9:951636. doi: 10.3389/fmolb.2022.951636 (PMC9519056; doi:10.3389/fmolb.2022.951636)
Supplement: Supplementary file 12 [file DataSheet5.PDF]

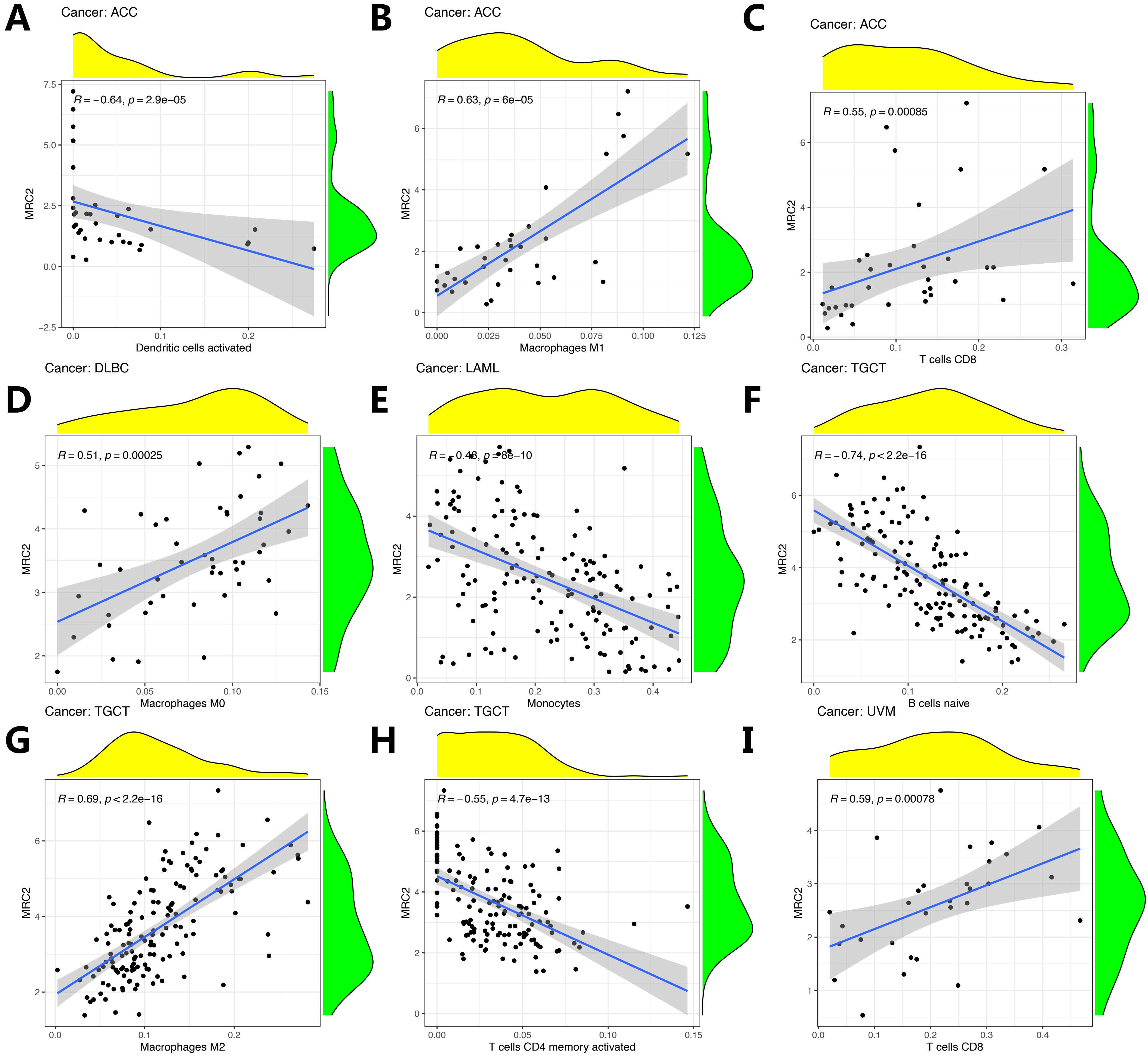

A
Cancer: ACC
R = −0.64, p = 2.9e−05
MRC2
Dendritic cells activated
B
Cancer: ACC
R = 0.63, p = 6e−05
MRC2
Macrophages M1
C
Cancer: ACC
R = 0.55, p = 0.00085
MRC2
T cells CD8
D
Cancer: DLBC
R = 0.51, p = 0.00025
MRC2
Macrophages M0
E
Cancer: LAML
R = −0.48, p = 8e−10
MRC2
Monocytes
F
Cancer: TGCT
R = −0.74, p < 2.2e−16
MRC2
B cells naive
G
Cancer: TGCT
R = 0.69, p < 2.2e−16
MRC2
Macrophages M2
H
Cancer: TGCT
R = −0.55, p = 4.7e−13
MRC2
T cells CD4 memory activated
I
Cancer: UVM
R = 0.59, p = 0.00078
MRC2
T cells CD8
