# Supplementary material for: Prognostic and immunotherapeutic significance of mannose receptor C type II in 33 cancers: An integrated analysis
Source: Front Mol Biosci. 2022 Sep 14;9:951636. doi: 10.3389/fmolb.2022.951636 (PMC9519056; doi:10.3389/fmolb.2022.951636)
Supplement: Supplementary file 17 [file DataSheet10.PDF]

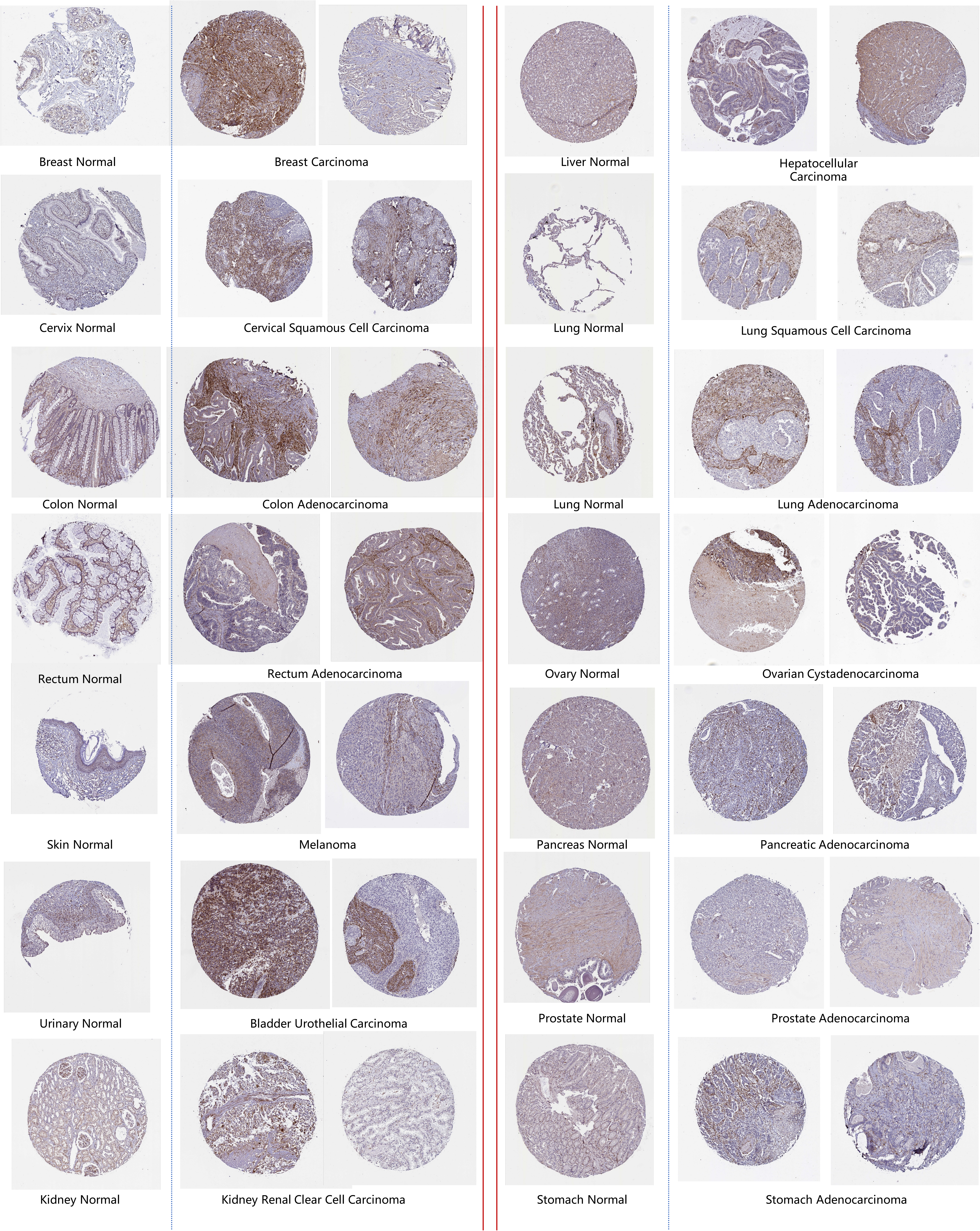
Breast Normal
Breast Carcinoma
Liver Normal
Hepatocellular Carcinoma
Cervix Normal
Cervical Squamous Cell Carcinoma
Lung Normal
Lung Squamous Cell Carcinoma
Colon Normal
Colon Adenocarcinoma
Lung Normal
Lung Adenocarcinoma
Rectum Normal
Rectum Adenocarcinoma
Ovary Normal
Ovarian Cystadenocarcinoma
Skin Normal
Melanoma
Pancreas Normal
Pancreatic Adenocarcinoma
Urinary Normal
Bladder Urothelial Carcinoma
Prostate Normal
Prostate Adenocarcinoma
Kidney Normal
Kidney Renal Clear Cell Carcinoma
Stomach Normal
Stomach Adenocarcinoma
